# Neurobehavioral Disorders and Cognitive Impairment in Methcathinone Exposure: A Systematic Review of Literature

**DOI:** 10.2174/011570159X387589250318041633

**Published:** 2025-03-25

**Authors:** Yihan Wang, Ning Wang, Shuquan Zhao

**Affiliations:** 1 Department of Forensic Pathology, Guangdong Province Translational Forensic Medicine Engineering Technology Research Center, Zhongshan School of Medicine, Sun Yat-sen University, Guangzhou, China

**Keywords:** Methcathinone, brain injury, adult offspring, developmental harm, cognitive impairment, neurobehavioral tests

## Abstract

**Background:**

Methcathinone, a synthetic cathinone derivative similar to amphetamine, has transitioned from a 1920s ephedrine precursor and Soviet-era antidepressant to a recreationally used substance since the 1970s-1980s, raising public health concerns due to its addiction potential and neurotoxicity-related health risks.

**Objective:**

This review comprehensively analyzes methcathinone's impact on adult offspring, synthesizing recent advancements and critiquing literature to pinpoint key findings, challenges, and future research directions.

**Methods:**

The systematic review adhered to PRISMA guidelines and encompassed case series, prospective and retrospective studies, as well as short communications published in English. An electronic search was conducted on PubMed, Elsevier, and CNKI. The focus was on methcathinone and its neuropsychological disorders and physical health complications, specifically in adult offspring.

**Results:**

A total of 8 studies met the inclusion criteria, resulting in a dataset of methcathinone on neurobehavioral and cognitive functions. These studies mainly found that prenatal methcathinone exposure in rats led to delayed physical development and induced anxiety-like behavior in offspring, with changes observed in neurobehavioral tests and the concentration of serotonin and dopamine. Furthermore, neurochemical effects were identified, showing dose- and time-dependent increases in extracellular dopamine and serotonin concentrations, and neurotoxic potential towards brain dopamine neurons.

**Conclusion:**

This study concludes that methcathinone poses severe risks, including neurotoxicity for users and developmental harm for offspring, necessitating ongoing research to comprehend associated risks and inform public health interventions.

## 1 INTRODUCTION

Methcathinone, also known as ephedrone, is a synthetic derivative of cathinone that possesses a chemical structure and pharmacological effects akin to those of amphetamine. Initially synthesized by German and French chemists in the late 1920s as a precursor in the production of ephedrine, methcathinone was later employed in the Soviet Union from the 1930s to the 1940s as an antidepressant before gaining popularity for recreational use, particularly in the 1970s and 1980s. It has emerged as a significant public health concern due to its high potential for addiction and severe neurotoxic effects. The substance has been linked to various adverse health outcomes, including neuropsychological disorders and physical health complications.

One of the primary harms associated with methcathinone use is its addictive potential. Research indicates that methcathinone exhibits a high propensity for abuse, comparable to other stimulants like methamphetamine and MDMA [1, 2]. Users often engage in binge patterns of consumption, which can lead to severe psychological effects, including paranoid psychosis and withdrawal symptoms [1, 3].

**Fig. (1):**
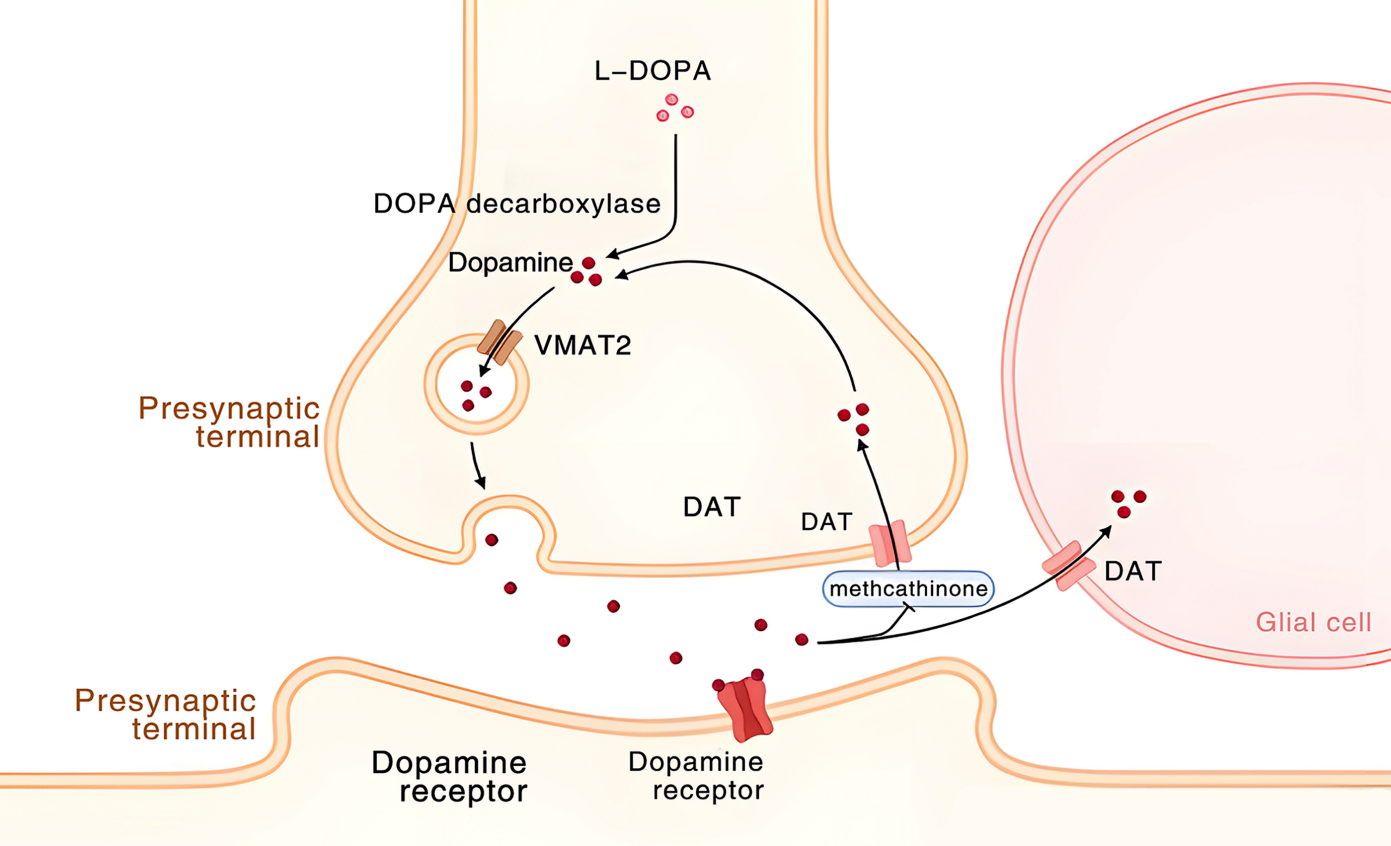
Methcathinone inhibits the reuptake of neurotransmitters by the DAT transporter, resulting in an increased concentration of neurotransmitters in the synaptic cleft.

Research suggests that methcathinone, akin to stimulants like methamphetamine and MDMA, can trigger neuropharmacological changes, including the damage of dopamine nerve endings in the striatum and disruptions in dopamine (Fig. **1**) and serotonin transporter functions, potentially leading to lasting neurotransmitter system deficiencies [4, 5]. Moreover, its liability for abuse is highlighted by the euphoria it induces, fueling repeated use and the risk of addiction. This neurotoxicity is intensified when the drug is commonly cut with other substances, amplifying its detrimental impacts and complicating treatment efforts [6, 7].

Moreover, methcathinone use has been linked to serious physical health issues, including manganese poisoning and parkinsonism. Reports have documented cases of manganese encephalopathy in individuals who inject homemade methcathinone, highlighting the severe neurological consequences of its use [8, 9]. The symptoms of this condition can include motor dysfunction and cognitive decline, which are particularly debilitating for users [9, 10]. Additionally, the cardiovascular effects of methcathinone, such as tachycardia and hypertension, pose immediate health risks that can lead to fatal outcomes [2, 11].

The social implications of methcathinone use are also significant. The drug's prevalence among vulnerable populations, including adolescents and intravenous drug users, raises concerns about the potential for increased rates of HIV and other infectious diseases due to risky behaviors associated with drug use [12]. Furthermore, the clandestine nature of its production and distribution complicates public health responses, as users often lack awareness of the specific risks associated with methcathinone and its derivatives [7, 13].

The objective of this review is to provide a comprehensive analysis of the effect of methcathinone on adult offspring, aiming to consolidate the progress made in recent years and offer a synthesized perspective on the current state of the field. By critically evaluating the existing literature, this review seeks to highlight the key developments, challenges, and opportunities within methcathinone affecting adult offspring while also identifying potential research gaps and areas for future exploration and guiding the direction for subsequent research and application in the field.

## 2 METHODS

**Fig. (2):**
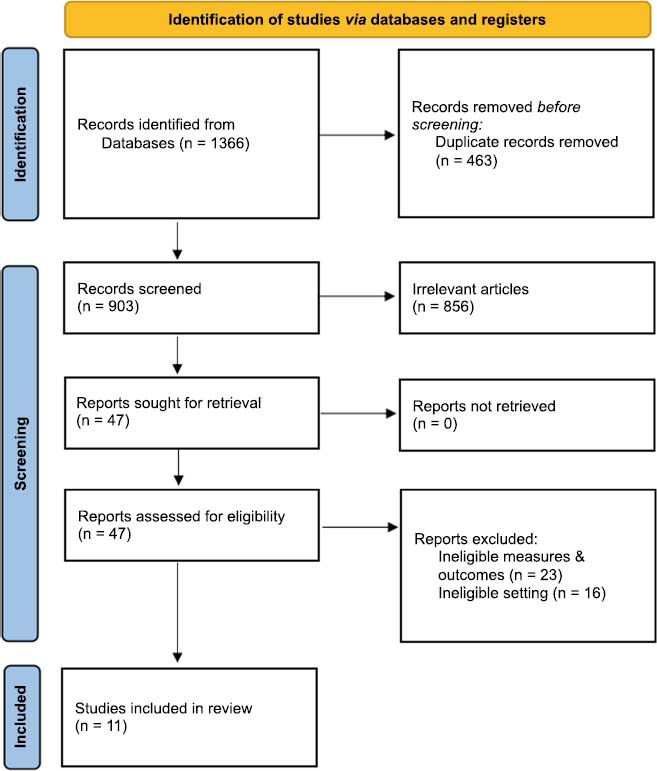
PRISMA 2020 flow diagram for this systematic review.

This review adheres to the PRISMA (Preferred Reporting Items for Systematic Reviews and Meta-Analyses) guidelines (Fig. **2**). In terms of the search strategy, a comprehensive electronic search was conducted across Pubmed, CNKI, and Elsevier databases from their inception until December 20, 2023. The search encompassed publications in all languages, using terms such as “methcathinone,” “ephedrone,” “cathinone,” “effect,” “offspring,” and “harm” in titles, abstracts, and keywords. To ensure the identification and evaluation of all relevant literature, reference lists of the located articles were also scrutinized. We meticulously reviewed all titles from each database and selected those with potential relevance, amounting to 47. The absence of language bias, as the search was not confined to English-language studies, minimized the risk of missing pertinent studies. Furthermore, no significant publication bias was detected, and the search did not yield any unpublished studies.

## 3 RESULTS

Eleven studies met the inclusion criteria (Fig. **2**), with eight contributing data on the impact of methcathinone on neurobehavioral and cognitive functions (Fig. **1**). However, three studies were excluded from the analysis as they did not delineate the precise neural pathways through which methcathinone influences neurobehavioral and cognitive outcomes.

The following data were extracted from the included studies, summarizing in Tables **1** and **2** the method and species, examined element, the main result of the study, and the study source.

This literature provides comprehensive insights into the multifaceted impacts of methcathinone exposure, particularly focusing on its prenatal and postnatal effects.

Youyou *et al*. conducted a comprehensive study examining the effects of methcathinone exposure during both prenatal and lactational periods on rat offspring. Their findings revealed that exposure resulted in notable deficits in learning and memory abilities, as assessed through various behavioral tests [14]. The study highlighted that these impairments were not only immediate but also persisted into later stages of development, suggesting long-lasting consequences of methcathinone exposure. The neurotoxic effects observed were attributed to alterations in neurodevelopmental processes, including neurogenesis and synaptic plasticity, which are critical for cognitive functions.

Further research has corroborated these findings, emphasizing the broader implications of methcathinone exposure on neurodevelopment. For instance, studies have shown that exposure to methcathinone can lead to alterations in neurotransmitter systems, particularly those involving dopamine and serotonin, which are crucial for mood regulation and cognitive functions. The disruption of these systems during critical periods of brain development can result in behavioral anomalies and cognitive deficits that manifest as the offspring mature. Additionally, it was observed to affect neurological behavior in the adult offspring, with serotonin and dopamine potentially playing a role in this process [15].

Moreover, the implications of these findings extend beyond animal models. The parallels drawn between rodent studies and human developmental outcomes suggest that maternal use of methcathinone could pose significant risks to human offspring, particularly in terms of neurodevelopmental disorders and cognitive impairments. This concern is compounded by the increasing prevalence of methcathinone use in certain populations, necessitating further investigation into its long-term effects on child development.

In addition to the aforementioned, the literature in question yields the following outcomes: 1) Neurochemical Alterations: Research on para-substituted methcathinone analogs reveals abuse-related neurochemical effects, notably affecting dopamine and serotonin levels in the nucleus accumbens. These findings underscore the complex interplay between substance use and neurotransmitter systems [14, 16-24]. 2) Parkinsonism and Manganese Toxicity: A notable finding is the association between methcathinone use and Parkinsonian syndrome, potentially linked to manganese toxicity [25]. This emphasizes the importance of considering metal toxicities in the context of substance-induced neurodegeneration. 3) Structural Brain Abnormalities: Grey matter alterations observed in methcathinone abusers with Parkinsonian symptoms highlight structural brain changes, further supporting the notion of lasting neural damage due to chronic exposure [26]. 4) Cognitive Impairment: Patients with manganese-methcathinone encephalopathy exhibit distinct cognitive profiles, reflecting widespread cognitive deficits. This underscores the need for targeted interventions to address these impairments [27].

## 4 DISCUSSION

The dangers of methcathinone, a potent stimulant drug, are multifaceted, encompassing neurotoxicity, potential harm to offspring, and ongoing research into its effects. Methcathinone, structurally related to amphetamines, exhibits significant neurotoxic properties, particularly affecting dopaminergic and serotonergic systems.

Studies have demonstrated that methcathinone can lead to persistent reductions in dopamine transporter (DAT) density, which is indicative of neurotoxicity in central nervous system (CNS) neurons [13, 16]. This reduction in DAT density is indicative of dopaminergic terminal loss, which has been observed in imaging studies of abstinent methcathinone users [17]. Additionally, the neurotoxic effects of methcathinone are compounded by its structural similarities to other neurotoxic substances, such as methamphetamine, which also leads to significant dopaminergic neurotoxicity [18]. The acute neurotoxic effects of methcathinone have been linked to the development of Parkinsonian symptoms in users, characterized by bradykinesia, rigidity, and dystonia [19].

Furthermore, the acute toxicity of methcathinone has been linked to hyperthermia, which exacerbates its neurotoxic effects by impairing mitochondrial function and increasing the generation of reactive oxygen species (ROS). This mitochondrial dysfunction is critical, as it can lead to neurodegenerative conditions similar to those seen in Parkinson's disease, highlighting the long-term risks associated with methcathinone use [16, 20, 21].

Limited evidence exists on the effects of methcathinone exposure during pregnancy, but findings suggest potential risks to fetal and neonatal development. Animal studies indicate that chronic prenatal and lactational exposure to methcathinone can delay physical development, disrupt neurological reflexes in offspring, and impair learning and memory abilities during adolescence. The delayed development of physical features, such as hair growth and neurological reflexes, observed in these animal studies indicates methcathinone exposure may interfere with normal fetal and neonatal development. The observed deficits in spatial learning, memory, and novel object exploration in adolescent offspring suggest prenatal and early postnatal methcathinone exposure can have lasting impacts on the brain and cognitive function. While human data is sparse, the limited evidence indicates prenatal methcathinone exposure may increase risks of adverse pregnancy outcomes like low birth weight, preterm delivery, and neonatal withdrawal symptoms. This is likely due to methcathinone's vasoconstrictive effects, which can disrupt fetal growth and development in a similar manner to other stimulants like cocaine [22].

Overall, the available data, though limited, suggests chronic in-utero and early-life exposure to methcathinone may pose significant risks to the developing fetus and child. Careful monitoring of fetal growth and neonatal health is warranted in cases of maternal methcathinone use, and avoidance of methcathinone during pregnancy is recommended, given the potential for lasting harm to the offspring. Further research is still needed to fully characterize the reproductive and developmental effects of methcathinone, but the existing evidence underscores the importance of preventing maternal exposure during critical periods of fetal and infant development.

The challenges in combating the dangers of methcathinone are significant, but they can be addressed through a targeted strategy. Research must be intensified to understand the long-term effects and develop treatments that counteract neurotoxicity and mitochondrial damage [28]. Public health campaigns should be implemented to educate at-risk groups about the drug’s risks, as noted by the importance of prevention strategies in reducing drug abuse [29]. Access to addiction services must be expanded to support recovery, a point emphasized by the need for comprehensive treatment approaches in substance abuse [30]. Regulatory controls need to be strengthened to reduce the drug’s availability, as highlighted in the literature on the effectiveness of supply reduction policies. Training for healthcare professionals is also essential to ensure they can effectively manage methcathinone-related issues, as discussed in the context of professional development in addiction medicine. By integrating these measures, we can mitigate the drug’s impact and safeguard public health.

## CONCLUSION

In conclusion, methcathinone poses significant dangers not only to individual users through its neurotoxic effects but also to future generations through potential developmental harm. The interplay between its pharmacological properties and the broader implications for health necessitates continued research to fully understand the risks associated with this substance and to inform public health strategies aimed at mitigating its impact.

## Figures and Tables

**Table 1:** Animal experiments.

**S. No.**	**Title**	**Method and Species**	**Examined Element**	**Main Results**
1	Effects of methcathinone exposure during prenatal and lactational periods on the development and the learning and memory abilities of rat offspring	Rat offspring rat	Morris Water Maze (MWM) test novel object recognition test	Neurobehavioral tests of the offspring rat Neurobehavioral abnormalities
2	Effects of prenatal methcathinone exposure on the neurological behavior of adult offspring	Rat offspring rat	Neurobehavioral tests open-field test Morris water maze test	The concentration of 5-HT and DA in the serum of rat pups
3	Abuse-related neurochemical effects of para-substituted methcathinone analogs in rats: Microdialysis studies of nucleus accumbens dopamine and serotonin	Rat	Microdialysis	Extracellular DA and 5-HT concentrations in each brain region
4	Discriminative and locomotor effects of five synthetic cathinones in rats and mice	Mice	Standard behavior-testing chambers	Maximal stimulation of locomotor activity
5	Neurotoxic and pharmacologic studies on enantiomers of the N-methylated analog of cathinone (methcathinone): A new drug of abuse	Mice & rats	Measure locomotor stimulation	Neurotoxic potential toward brain dopamine (DA)

**Table 2:** Human experiments.

**S. No.**	**Title**	**Method and Species**	**Examined Element**	**Main Results**
1	A Parkinsonian syndrome in methcathinone users and the role of manganese	23 patients (20 men and 3 women)	Unified Parkinson’s Disease Rating Scale (UPDRS)	HIV and hepatitis C antibodies, CD4 lymphocyte counts, liver function, serum ceruloplasmin, and copper levels
2	Grey matter abnormalities in methcathinone abusers with Parkinsonian syndrome	methcathinone abusers	T1-weighted structural and resting-state functional MRI	subcortical grey matter, putamen and thalamus bilaterally, and the left caudate nucleus
3	Cognitive profile of patients with manganese-methcathinone encephalopathy	patients with Manganese-methcathinone encephalopathy (MME)	Unified Parkinson’s Disease Rating Scale (UPDRS)	Parts I (mentation, behavior, and mood), II (activities of daily living; ADL), and III (motor examination) contain 44 questions

## LIST OF ABBREVIATIONS

CNS: Central Nervous System
DAT: Dopamine Transporter
PRISMA: Preferred Reporting Items for Systematic Reviews and Meta-Analyses
ROS: Reactive Oxygen Species

## STANDARDS OF REPORTING

PRISMA methodology and guidelines were followed.

## SUPPLEMENTARY MATERIAL

PRISMA checklist is available as supplementary material on the publisher’s website along with the published article.

## References

[1] Rojek S., Kłys M., Maciów-Głąb M., Kula K., Strona M. (2014). Cathinones derivatives‐related deaths as exemplified by two fatal cases involving methcathinone with 4‐methylmethcathinone and 4‐methylethcathinone.. Drug Test. Anal..

[2] Eshleman A.J., Wolfrum K.M., Hatfield M.G., Johnson R.A., Murphy K.V., Janowsky A. (2013). Substituted methcathinones differ in transporter and receptor interactions.. Biochem. Pharmacol..

[3] Miliano C., Serpelloni G., Rimondo C., Mereu M., Marti M., De Luca M.A. (2016). Neuropharmacology of new psychoactive substances (nps): Focus on the rewarding and reinforcing properties of cannabimimetics and amphetamine-like stimulants.. Front. Neurosci..

[4] Hadlock G.C., Webb K.M., McFadden L.M., Chu P.W., Ellis J.D., Allen S.C., Andrenyak D.M., Vieira-Brock P.L., German C.L., Conrad K.M., Hoonakker A.J., Gibb J.W., Wilkins D.G., Hanson G.R., Fleckenstein A.E. (2011). 4-Methylmethcathinone (mephedrone): Neuropharmacological effects of a designer stimulant of abuse.. J. Pharmacol. Exp. Ther..

[5] Pantano F., Tittarelli R., Mannocchi G., Pacifici R., di Luca A., Busardò F.P., Marinelli E. (2017). Neurotoxicity induced by mephedrone: An up-to-date review.. Curr. Neuropharmacol..

[6] Pascoe M.J., Radley S., Simmons H.T.D., Measham F. (2022). The cathinone hydra: Increased cathinone and caffeine adulteration in the english MDMA market after brexit and covid-19 lockdowns.. Drug Sci. Policy Law.

[7] Guirguis A., Corkery J.M., Stair J.L., Kirton S.B., Zloh M., Schifano F. (2017). Intended and unintended use of cathinone mixtures.. Hum. Psychopharmacol..

[8] Habrat B., Silczuk A., Klimkiewicz A. (2021). Manganese encephalopathy caused by homemade methcathinone (ephedrone) prevalence in poland.. Nutrients.

[9] de Bie R.M.A., Gladstone R.M., Strafella A.P., Ko J.H., Lang A.E. (2007). Manganese-induced parkinsonism associated with methcathinone (Ephedrone) abuse.. Arch. Neurol..

[10] Koziorowski D., Szlufik S., Mandat T., Kłoda M., Duszyńska-Wąs K., Drzewinska A., Friedman A. (2016). Improvement in ephedrone parkinsonism after global pallidus pars interna deep brain stimulation implantation.. Mov. Disord. Clin. Pract..

[11] Smith D., Negus S., Blough B., Banks M. (2016). Cocaine‐like discriminative stimulus effects of amphetamine, cathinone, and alpha‐pyrrolidinovalerophenone analogs in male rhesus monkeys.. FASEB J..

[12] Busza J.R., Balakireva O.M., Teltschik A., Bondar T.V., Sereda Y.V., Meynell C., Sakovych O. (2011). Street-based adolescents at high risk of HIV in Ukraine.. J. Epidemiol. Community Health.

[13] Brunt T.M., Poortman A., Niesink R.J.M., van den Brink W. (2011). Instability of the ecstasy market and a new kid on the block.. Mephedrone. J. Psychopharmacol.

[14] Youyou Z., Yalei Y., Yanfei D., Shuquan Z., Zhaoyang L., Liang R., Liang L. (2020). Effects of methcathinone exposure during prenatal and lactational periods on the development and the learning and memory abilities of rat offspring.. Neurotox. Res..

[15] Youyou Z., Zhaoyang L., Chen L., Shuquan Z., Hui W. (2024). Effects of prenatal methcathinone exposure on the neurological behavior of adult offspring.. Curr. Neuropharmacol..

[16] Stepens A., Groma V., Skuja S., Platkājis A., Aldiņš P., Ekšteina I., Mārtiņsone I., Bricis R., Donaghy M. (2014). The outcome of the movement disorder in methcathinone abusers: Clinical, MRI and manganesemia changes, and neuropathology.. Eur. J. Neurol..

[17] Angoa-Pérez M., Kane M.J., Francescutti D.M., Sykes K.E., Shah M.M., Mohammed A.M., Thomas D.M., Kuhn D.M. (2012). Mephedrone, an abused psychoactive component of ‘bath salts’ and methamphetamine congener, does not cause neurotoxicity to dopamine nerve endings of the striatum.. J. Neurochem..

[18] Anneken J.H., Angoa-Pérez M., Sati G.C., Crich D., Kuhn D.M. (2018). Assessing the role of dopamine in the differential neurotoxicity patterns of methamphetamine, mephedrone, methcathinone and 4-methylmethamphetamine.. Neuropharmacology.

[19] Selikhova M., Fedoryshyn L., Matviyenko Y., Komnatska I., Kyrylchuk M., Królicki L., Friedman A., Taylor A., Jäger H.R., Lees A., Sanotsky Y. (2008). Parkinsonism and dystonia caused by the illicit use of ephedrone—A longitudinal study.. Mov. Disord..

[20] Zhou X., Bouitbir J., Liechti M.E., Krähenbühl S., Mancuso R.V. (2020). Hyperthermia increases neurotoxicity associated with novel methcathinones.. Cells.

[21] Zhou X., Bouitbir J., Liechti M.E., Krähenbühl S., Mancuso R.V. (2020). Para-halogenation of amphetamine and methcathinone increases the mitochondrial toxicity in undifferentiated and differentiated sh-sy5y cells.. Int. J. Mol. Sci..

[22] Suyama J.A., Sakloth F., Kolanos R., Glennon R.A., Lazenka M.F., Negus S.S., Banks M.L. (2016). Abuse-related neurochemical effects of para-substituted methcathinone analogs in rats: Microdialysis studies of nucleus accumbens dopamine and serotonin.. J. Pharmacol. Exp. Ther..

[23] Gatch M.B., Rutledge M.A., Forster M.J. (2015). Discriminative and locomotor effects of five synthetic cathinones in rats and mice.. Psychopharmacology..

[24] Sparago M., Wlos J., Yuan J., Hatzidimitriou G., Tolliver J., Dal Cason T.A., Katz J., Ricaurte G. (1996). Neurotoxic and pharmacologic studies on enantiomers of the N-methylated analog of cathinone (methcathinone): A new drug of abuse.. J. Pharmacol. Exp. Ther..

[25] Stepens A., Logina I., Liguts V., Aldiņš P., Ekšteina I., Platkājis A., Mārtiņsone I., Tērauds E., Rozentāle B., Donaghy M. (2008). A Parkinsonian syndrome in methcathinone users and the role of manganese.. N. Engl. J. Med..

[26] Juurmaa J., Menke R.A.L., Vila P., Müürsepp A., Tomberg T., Ilves P., Nigul M., Johansen-Berg H., Donaghy M., Stagg C.J., Stepens A., Taba P. (2016). Grey matter abnormalities in methcathinone abusers with a Parkinsonian syndrome.. Brain Behav..

[27] Ennok M., Sikk K., Haldre S., Taba P. (2020). Cognitive profile of patients with manganese-methcathinone encephalopathy.. Neurotoxicology.

[28] Meyer M.R., Maurer H.H. (2010). Metabolism of designer drugs of abuse: An updated review.. Curr. Drug Metab..

[29] Prevention programmes (2018). https://www.drugabuse.gov/publications/drugfacts/prevention-programs.

[30] McCance-Katz E.F. (2018). The substance abuse and mental health services administration (SAMHSA): New directions.. Psychiatr. Serv..

